# Goats as sentinel hosts for the detection of tick-borne encephalitis risk areas in the Canton of Valais, Switzerland

**DOI:** 10.1186/s12917-017-1136-y

**Published:** 2017-07-11

**Authors:** Nadia Rieille, Christine Klaus, Donata Hoffmann, Olivier Péter, Maarten J. Voordouw

**Affiliations:** 1 Central Institute of Valais Hospitals, Infectious diseases, Av Grand Champsec 86, -1950 Sion, CH Switzerland; 2 Friedrich-Loeffler-Institut, Institute of Bacterial Infections and Zoonoses, Naumburger Str. 96a, D-07743 Jena, Germany; 3 Friedrich-Loeffler-Institut, Institute of Diagnostic Virology, Südufer 10, D-17493 Greifswald-Insel Riems, Germany; 4 Institute of Biology, Laboratory of Ecology and Evolution of parasites, University of Neuchâtel, Rue Emile-Argand 11, 2000 Neuchâtel, Neuchâtel Switzerland

**Keywords:** ELISA, Flavivirus, Goats, *Ixodes ricinus*, Sentinel host, Seroprevalence, Switzerland, Tick-borne encephalitis virus, Vector-borne disease

## Abstract

**Background:**

Tick-borne encephalitis (TBE) is an important tick-borne disease in Europe. Detection of the TBE virus (TBEV) in local populations of *Ixodes ricinus* ticks is the most reliable proof that a given area is at risk for TBE, but this approach is time-consuming and expensive. A cheaper and simpler approach is to use immunology-based methods to screen vertebrate hosts for TBEV-specific antibodies and subsequently test the tick populations at locations with seropositive animals.

**Results:**

The purpose of the present study was to use goats as sentinel animals to identify new risk areas for TBE in the canton of Valais in Switzerland. A total of 4114 individual goat sera were screened for TBEV-specific antibodies using immunological methods. According to our ELISA assay, 175 goat sera reacted strongly with TBEV antigen, resulting in a seroprevalence rate of 4.3%. The serum neutralization test confirmed that 70 of the 173 ELISA-positive sera had neutralizing antibodies against TBEV. Most of the 26 seropositive goat flocks were detected in the known risk areas in the canton of Valais, with some spread into the connecting valley of Saas and to the east of the town of Brig. One seropositive site was 60 km to the west of the known TBEV-endemic area. At two of the three locations where goats were seropositive, the local tick populations also tested positive for TBEV.

**Conclusion:**

The combined approach of screening vertebrate hosts for TBEV-specific antibodies followed by testing the local tick population for TBEV allowed us to detect two new TBEV foci in the canton of Valais. The present study showed that goats are useful sentinel animals for the detection of new TBEV risk areas.

**Electronic supplementary material:**

The online version of this article (doi:10.1186/s12917-017-1136-y) contains supplementary material, which is available to authorized users.

## Background

Tick-borne encephalitis (TBE) is the most important viral tick-borne zoonosis in Europe and causes between 5352 (in 2008) and 12,733 (in 1996) human cases per year in Europe and parts of Asia, especially in the Siberian part of Russia [1]. In Switzerland, where TBE has been treated as a notifiable disease since 1984 [2], about 100–130 cases are reported each year (the maximum was 244 cases in 2006). The reasons for these annual fluctuations in the incidence of TBE are not well understood. Across Europe, geographic variation in the prevalence of TBE in humans is largely dependent on climate factors that influence the questing activity of ticks [3]. In addition, anthropomorphic changes in agriculture and outdoor and leisure activities influence the risk that humans will contract TBE [4]. Theoretical models that examine how climate change will influence tick ecology predict that the TBE virus (TBEV) will spread to the north and to higher altitudes over the next decades [5]. Field studies have confirmed that TBEV has spread northwards in Norway [6] and to higher altitudes in the Czech Republic and Austria [7–10]. The spread of TBEV to higher altitudes is also an important concern in Switzerland.

TBEV is a member of the Flavivirus genus that includes the yellow fever virus and the dengue virus [11]. In Central Europe, the main vector for TBEV is the hard tick *Ixodes ricinus*, which has three blood-feeding stages: larva, nymph, and adult. The larvae and nymphs maintain TBEV in nature because they feed on the same group of TBEV-competent reservoir hosts, mainly wild rodents [5]. Larval ticks acquire the virus after feeding on an infected rodent, but this mode of transmission is relatively inefficient because the duration of infectivity to ticks is short (2-3 days) [12]. Other studies have shown that TBEV can be found in rodent tissues at 10 to 50 days post-infection [13]. Compared to other tick-borne pathogens, the prevalence of TBEV in *I. ricinus* populations is generally very low (< 1.0%) [14, 15]. Theoretical models have shown that the short duration of infectivity is the main reason why TBEV has such a low prevalence in nature [16–18]. Larval ticks can also acquire TBEV by co-feeding transmission where they feed in close proximity to an infected nymph on the same reservoir host [12, 18, 19]. Co-feeding transmission is a fragile mode of transmission because it depends on the synchronized questing activity of larval and nymphal ticks, which in turn, depend on a particular set of climatic conditions [5]. This particular set of climatic conditions is one reason why TBEV has a patchy geographic distribution across Europe [5]. Even in areas where TBEV is endemic, the presence of the virus in ticks and reservoir hosts is often highly focal [20, 21]. Pawlovskij pointed out that a natural TBEV focus depends on a number of botanical, zoological, climatical and geo-ecological conditions [22].

In veterinary medicine, clinical cases of TBE are rare, but have been reported in horses [23] and dogs [24, 25]. Other species like goats, sheep and cattle develop antibody titres without exhibiting clinical signs. These species are of high relevance for the so-called alimentary TBE. During viraemia, TBEV is excreted into the milk and can be ingested via consumption of raw milk or raw milk products such as cheese. While TBE in humans is mostly caused by tick bites, cases of alimentary TBE have been reported in recent years from Slovakia [26], Estonia [27], the Czech Republic [28], Austria [9], and Hungary [29].

Many studies have surveyed populations of wild *I. ricinus* ticks for the prevalence of TBEV [30–32]. The advantage of this approach is the direct detection of the virus in the tick vector. The disadvantage is that testing ticks for TBEV is time-consuming and expensive. Due to the low prevalence of TBEV in ticks (0.1–5.0% [14, 15]), and the high variability of TBEV prevalence in space and time, there is much interest in developing alternative methods to assess the human risk of TBE [33]. One such method is the detection of TBEV-specific antibodies in sentinel vertebrate hosts. Various wild and domestic vertebrates such as rodents [13, 34, 35], roe deer [36], goats [37, 38], sheep [39], dogs [40, 41], and even brown bears [42] have been used as sentinels in endemic areas. A disadvantage of this method is that detection of antibodies does not provide any information on the time and place of infection. An advantage of this method is that vertebrate hosts can feed many ticks and therefore “amplify” the TBEV signal in a given area. Another advantage is that screening vertebrate serum samples for TBEV-specific antibodies is fast and cheap. Goats, sheep and horses may be especially well-suited as sentinel hosts because they graze in meadows over long periods each year and are therefore potentially exposed to many TBEV-infected ticks. In addition, small ruminants and horses are kept in locations that are well known to the owner, which provides information on the location of TBEV infection. These locations can then be sampled for ticks to test for the existence of an endemic TBEV focus [43, 44].

Since 2013, the Swiss Federal Office for Public Health (FOPH) has edited two different maps with respect to TBEV. One map shows the risk of TBE infection and is based on the frequency of human TBE cases. The other map indicates the areas where the FOPH recommends prophylactic vaccination against TBEV. This map describes a risk area by using all relevant information, including human cases of TBE and TBEV-positive ticks (Bull OFSP 18/2013). A recent area of interest in Switzerland with respect to TBEV is the canton of Valais, which is located in the south of Switzerland. In this canton, 17 human cases have been described within the past 10 years, 15 of them since 2010. In 2009, two foci of TBEV were identified in Valais by a large nation-wide survey that screened populations of *I. ricinus* ticks [30]. Over the following years, the presence of TBEV in these areas was confirmed. A large tick survey that sampled more than 19,000 ticks across the canton of Valais found four new foci close to the two original ones [45, 46]. The aim of the present study was to use goats as sentinels to confirm existing TBEV risk areas and to detect new ones. The value of goats as sentinel animals was confirmed by analyzing ticks collected from areas identified by sero-positive goats. Our study demonstrates that testing antibodies in goats is an effective method for detecting new foci of TBEV.

## Methods

### Serum collection from goats

Goat sera were collected as part of a national survey supervised by the Swiss Veterinary Service on caprine arthritis encephalitis (CAE), a viral disease that occurs exclusively in goats. A total of 4114 individual goat sera were collected between October 2011 and March 2012. Only goats older than 6 months were sampled. The Cantonal Veterinary Service of the Canton of Valais kindly provided us with these goat serum samples, which were used in the present study.

### ELISA procedure

We adapted the Serion ELISA classic TBE virus IgG (quantitative) test for humans (Serion GmbH, Germany) for veterinary use. Here solid phase compounds of the ELISA (coated plates) were used, while solutions were prepared in the laboratory (see below). The positive control was provided by the Institute of Bacterial Infections and Zoonoses, Friedrich-Loeffler-Institut, Jena, Germany. This goat was vaccinated four times at weeks 1, 2, 4, and 12 with FSME-Immun for adults (Baxter, Deutschland GmbH, Germany), as described by Klaus et al. [47]. The reagents for the ELISA protocol were prepared as follows: the dilution buffer contained 0.5% of BSA diluted in TBS solution, and the washing buffer consisted of TBS solution (pH 7.5) containing 0.5% Tween 20. For the secondary antibody, a rabbit-derived anti-goat immunoglobulin G conjugated to recombinant horseradish peroxidase (Invitrogen by Thermo Fischer Scientific, Rockford, IL 61105 USA) was used. This secondary antibody was used at a dilution of 1:7500 and the corresponding peroxidase substrate (Invitrogen) was added following the manufacturer’s instructions. The stopping solution was 0.5 M sulfuric acid.

All ELISA tests were performed using a DSX workstation (Dynex technologies, Worthing, UK). The following protocol was used: 200 μl of dilution buffer and 10 μl of serum corresponding to a dilution of 1:21 were added to each well and incubated at room temperature (20–25 °C) for 75 min. Wells were washed four times with 300 μl of washing buffer before adding 100 μl of the secondary antibody at room temperature for 25 min. Wells were washed four times with 300 μl of washing buffer before adding 100 μl of substrate solution. After incubating the plate for 5 min in the dark at room temperature, the reaction was stopped by the addition of 100 μl of H_2_SO_4_. The optical density (OD) was measured at 405 nm.

### Determination of threshold values

We established an optical density (OD) value that we considered as the threshold between TBEV-seropositive and seronegative goats. The data used to calculate the threshold were based on 200 serum samples from goats living in a part of the canton where the virus is presumed to be absent. The threshold was calculated as three standard deviations from the mean OD value (mean OD = 0.032, standard deviation = 0.34, threshold = 1.01). Goats with OD values >1 and <1 were considered seropositive and seronegative for TBEV, respectively.

### Serum neutralization test

All goat serum samples that tested positive in the ELISA (OD value >1) were retested using the serum neutralization test (SNT). The SNT is considered as the gold standard for establishing whether vertebrate hosts have been exposed to TBEV or not. In addition, we randomly selected 100 serum samples and tested them using SNT to demonstrate that the ELISA was an efficient prescreen for detecting TBEV-positive serum samples. The SNT described by Klaus et al. [37] was used: the avirulent TBEV strain Langat was used with 100 TCID_50_/well. The virus titre used was confirmed by back-titrations. Serum samples were inactivated by freezing and thawing as a first step and by incubating them at 56 °C for 30 min as a second step. They were titrated in duplicate starting at a dilution of 1:5 in MEM Earle’s medium. After an incubation period of 24 h at 37 °C, a BHK-21 cell suspension was added and incubated for four additional days. Virus replication was detected using immunofluorescence analysis and a TBEV-specific rabbit-antiserum. Titres were expressed as the reciprocal of the dilution that caused 50% neutralization (ND50).

### Absorption test

An absorption test (AT) was done for all serum samples that tested positive for the ELISA (OD value >1). To determine the optimal serum dilution for the AT, the OD values of 9 positive samples (OD > 1) and 6 borderline negative samples (0.8 < OD < 1) were retested over a range of dilutions: 1:100 to 1:25,600. The protocol for the AT included the creation of an absorbed mixture and a control mixture for each tested serum sample. The absorbed mixture was prepared by incubating 50 μl of 1:125 pre-diluted sera in the presence of 50 μl of 1:2 pre-diluted TBEV antigen (25 μl of TBE vaccine (FSME-Immun CC, Baxter) diluted in 25 μl of dilution buffer). The control mixture was prepared by incubating 50 μl of 1:125 pre-diluted sera in the presence of 50 μl of dilution buffer. The absorbed and control mixtures were incubated at room temperature for 3 h before performing the classical ELISA test described above. For each serum sample, we obtained an OD value for the control mixture (ODc) and the absorbed mixture (ODa) and calculated the extinction rate (Er) as follows: Er = 100*(ODc - ODa)/ODc. Serum samples with an Er value >55% and <45% were considered as seropositive and seronegative for TBEV antibodies, respectively. Serum samples exhibiting 45% < Er <55% were considered doubtful.

### Data collection

The names and residences of the goat owners were obtained from the Veterinary Office of the Canton of Valais. The Swiss Federation of Goat Rearing (SFGR) kindly provided additional information from member goat owners with respect to origin, sex, breed and age of the goats. All goat owners of SNT-positive animals and eight owners of SNT-negative flocks were contacted. Owners who agreed to participate in our study were asked where they pastured their goats. Owners who were not members of the SFGR were asked to provide information about their goats. The distances between the owners’ residences and the pasture sites were measured using the tools of Google Maps Labs.

### Tick collection

**Fig. 1:**
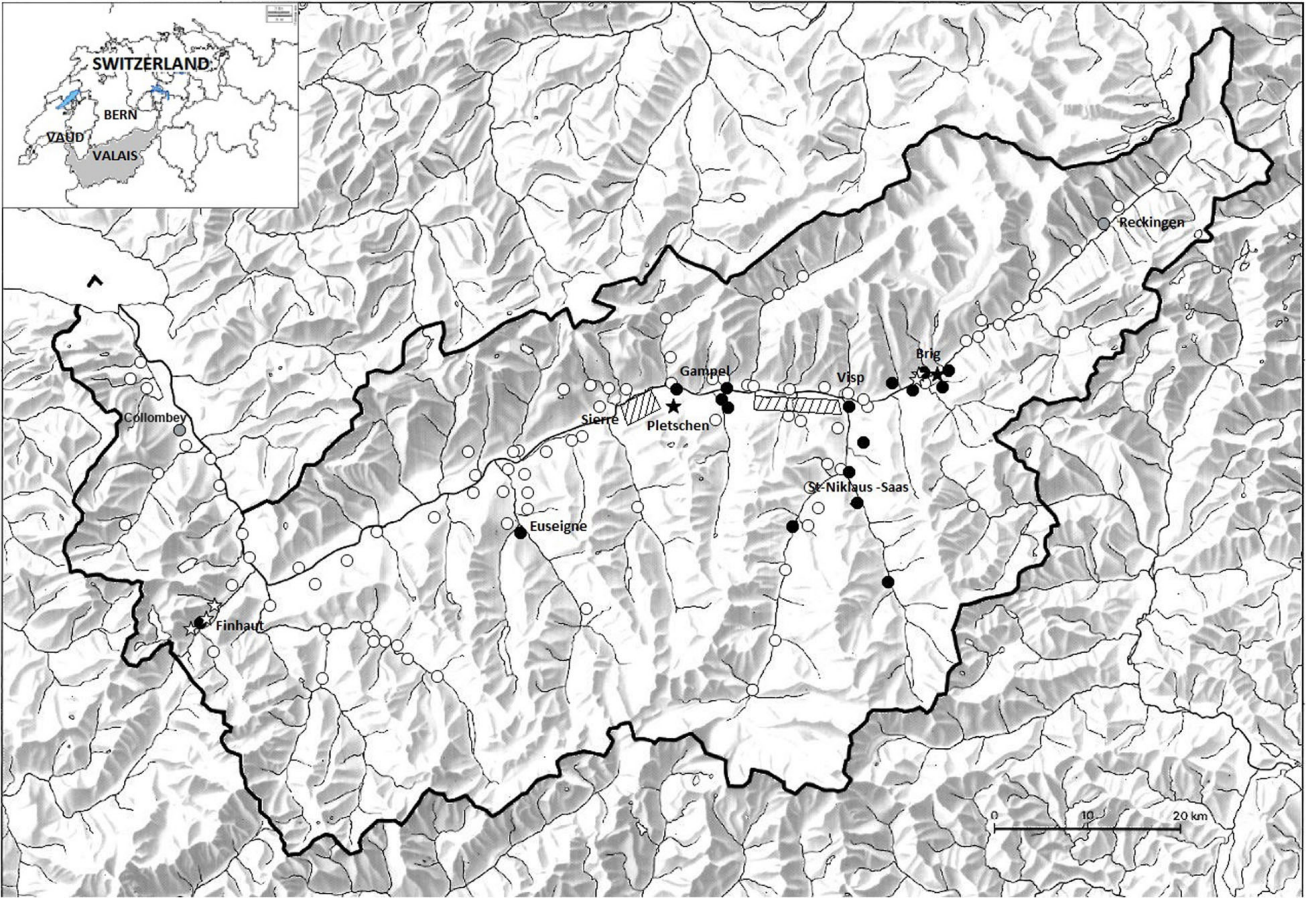
Location of the residences of the goat owners. seropositive goats, seronegative goats, imported seropositive goats from other Swiss cantons, TBE positive tick pool, TBE negative tick pool, known endemic areas for TBE virus

The detection of TBEV-seropositive flocks (see results) allowed us to select three sites in the canton of Valais that are potentially new TBEV foci. The three sites were located (1) near the town of Brig, (2) near the town of Gampel on the north side of the Rhone River, and (3) in an isolated area to the west of the municipality of Finhaut (Fig. 1). To confirm whether these three sites were true TBEV foci, *I. ricinus* ticks were sampled from the different pasture sites of these TBEV-seropositive flocks by dragging a white cotton flag over the ground in forested areas [45]. The ticks were frozen at −80 °C until use. For each sample site, ticks were identified and separated according to species, sex, and developmental stage. Pools of 10 adult ticks or 50 nymphs were combined for lysis and DNA/RNA extraction. Quantitative realtime RT-PCR was performed according to the method described by Gäumann et al. [30].

### Analysis of data

The serum neutralization test was considered as the gold standard for estimating the sensitivity and specificity of the ELISA and absorption test. Only serum samples confirmed by the serum neutralization test were used to create the maps of TBEV-positive goats.

## Results

### Goats

#### ELISA test

The 4114 goats examined in this study represent 73.7% (4114/5583) of all goats older than 6 months living in the canton of Valais. These goats belonged to 277 owners whose places of residence were distributed over 105 localities covering all 13 districts of the canton of Valais. Almost half of the goat serum samples (2048) were collected from two of these 13 districts (Brig and Visp) (Table 1). Of the 4114 goat serum samples, 175 (4.25%) samples were positive according to the ELISA test. The 175 sero-positive goats came from 88 different flocks. Of these 88 flocks, 55 had only one positive goat.

**Table 1:** Location of the goat flocks that were tested for IgG antibodies against the tick-borne encephalitis virus^a^

District	Location	Latitude	Longitude	n.flock	n.sera
Brig	Brig	46°18′60″ N	7°59′23″ E	1	8
	Eggerberg	46°18′25″ N	7°52′51″ E	1	14
	Glis	46°18′33″ N	7°58′21″ E	4	38
	Mund	46°18′59″ N	7°56′34″ E	12	250
	Naters	46°19′33″ N	7°59′17″ E	18	368
	Ried-Brig	46°18′44″ N	8°00′52″ E	2	13
	Simplon Dorf	46°11′45″ N	8°03′22″ E	2	6
	Termen	46°19′40″ N	8°01′20″ E	3	10
Total				43 (15.52%)	707 (17.19%)
Conthey	Nendaz	46°11′13″ N	7°18′10″ E	2	6
Total				2 (0.73%)	6 (0.15%)
Entremont	Bruson	46°03′57″ N	7°13′06″ E	1	7
	Fionnay	46°01′58″ N	7°18′27″ E	1	16
	Le Châble	46°04′50″ N	7°12′32″ E	3	18
	Lourtier	46°02′57″ N	7°15′57″ E	2	30
	Orsières	46°01′50″ N	7°08′47″ E	2	22
	Sarreyer	46°03′46″ N	7°15′03″ E	1	2
	Sembrancher	46°04′42″ N	7°09′02″ E	2	38
	Versegères	46°03′57″ N	7°14′02″ E	4	23
Total				16 (5.78%)	156 (3.﻿79%)
Goms	Binn	46°21′51″ N	8°11′02″ E	1	24
	Blitzingen	46°26′36″ N	8°12′08″ E	1	25
	Ernen	46°23′55″ N	8°08′45″ E	3	33
	Fieschertal	46°25′16″ N	8°08′34″ E	2	77
	Lax	46°23′20″ N	8°07′12″ E	1	13
	Münster	46°29′10″ N	8°15′44″ E	1	17
	Obergesteln	46°30′49″ N	8°19′25″ E	1	4
	Reckingen	46°28′10″ N	8°14′31″ E	2	103
Total				12 (4.33%)	296 (7.19%)
Hérens	Euseigne	46°10′19″ N	7°25′22″ E	2	57
	Hérémence	46°10′53″ N	7°24′16″ E	1	24
	La sage	46°05′55″ N	7°30′54″ E	3	20
	Mase	46°11′42″ N	7°25′60″ E	1	30
	Nax	46°13′42″ N	7°25′42″ E	2	14
	Vernamiège	46°12′38″ N	7°25′57″ E	2	69
	Vex	46°12′41″ N	7°23′53″ E	1	5
Total				12 (4.33%)	219 (5.32%)
Leuk	Albinen	46°20′31″ N	7°37′59″ E	1	66
	Bratsch	46°19′15″ N	7°42′26″ E	1	2
	Ergisch	46°17′35″ N	7°42′49″ E	1	6
	Erschmatt	46°19′17″ N	7°41′32″ E	2	27
	Gampel	46°18′56″ N	7°44′24″ E	1	22
	Leuk Stadt	46°19′03″ N	7°38′06″ E	3	25
	Leukerbad	46°22′47″ N	7°37′40″ E	2	11
	Niedergampel	46°18′45″ N	7°42′43″ E	3	41
	Oberems	46°16′54″ N	7°41′44″ E	2	13
	Salgesch	46°18′42″ N	7°34′14″ E	2	21
	Susten	46°18′39″ N	7°38′30″ E	8	66
	Turtmann	46°18′05″ N	7°42′16″ E	1	2
Total				27 (9.75%)	302 (7.34%)
Martigny	Charrat	46°07′19″ N	7°08′10″ E	3	8
	Fully	46°08′15″ N	7°06′51″ E	5	78
	Martigny	46°06′02″ N	7°04′26″ E	5	50
	Riddes	46°10′23″ N	7°13′21″ E	1	2
	Saxon	46°08′43″ N	7°10′49″ E	1	2
	Trient	46°03′22″ N	6°59′41″ E	2	16
Total				17 (6.14%)	156 (3.79%)
Monthey	Champéry	46°10′43″ N	6°52′12″ E	4	45
	Collombey	46°16′16″ N	6°56′44″ E	1	37
	Monthey	46°15′20″ N	6°57′17″ E	1	1
	Torgon	46°19′14″ N	6°52′36″ E	1	26
	Troistorrents	46°13′42″ N	6°54′58″ E	1	24
	Vionnaz	46°18′41″ N	6°53′58″ E	1	2
	Vouvry	46°20′11″ N	6°53′28″ E	1	3
Total				10 (3.61%)	138 (3.35%)
Oestlich Raron	Betten	46°22′35″ N	8°04′09″ E	3	15
	Bister	46°21′37″ N	8°03′53″ E	2	66
	Grengiols	46°22′20″ N	8°05′35″ E	3	29
	Mörel	46°21′23″ N	8°02′50″ E	4	93
Total				12 (4.33%)	203 (4.93%)
Sierre	Chalais	46°15′57″ N	7°30′32″ E	2	61
	Crans-Montana	46°18′41″ N	7°29′01″ E	1	2
	Grône	46°15′03″ N	7°27′31″ E	1	12
	Miège	46°18′43″ N	7°32′50″ E	1	2
	Mollens	46°18′56″ N	7°31′14″ E	2	5
	Réchy	46°15′41″ N	7°29′43″ E	2	14
	Sierre	46°17′39″ N	7°32′00″ E	4	29
	St-Jean	46°11′49″ N	7°35′09″ E	3	75
	St-Léonard	46°15′05″ N	7°25′11″ E	2	7
Total				18 (6.50%)	207 (5.03%)
Sion	Bramois	46°14′00″ N	7°24′20″ E	1	4
	Salins	46°12′38″ N	7°21′25″ E	1	8
	Sion	46°13′40″ N	7°21′33″ E	1	7
	St-Germain	46°15′01″ N	7°20′59″ E	2	25
	Uvrier	46°15′02″ N	7°24′45″ E	1	2
Total				6 (2.17%)	46 (1.12%)
St-Maurice	Collonges	46°10′14″ N	7°02′06″ E	1	3
	Dorénaz	46°08′50″ N	7°02′39″ E	5	33
	Finhaut	46°05′00″ N	6°58′37″ E	1	26
	Massongex	46°14′33″ N	6°59′22″ E	2	13
	Salvan	46°07′14″ N	7°01′15″ E	2	18
	St-Maurice	46°12′60″ N	7°00′07″ E	2	7
Total				13 (4.69%)	100 (2.43%)
Visp	Eisten	46°12′02″ N	7°53′36″ E	2	12
	Embd	46°12′54″ N	7°49′42″ E	2	33
	Eyholz	46°17′38″ N	7°54′32″ E	1	5
	Gasenried	46°10′43″ N	7°49′29″ E	1	18
	Grächen	46°11′43″ N	7°50′18″ E	8	76
	Herbriggen	46°08′07″ N	7°47′33″ E	3	76
	Lalden	46°18′01″ N	7°54′13″ E	3	39
	Saas-Grund	46°07′22″ N	7°56′11″ E	3	16
	St-Niklaus	46°10′40″ N	7°48′11″ E	23	542
	Stalden	46°13′59″ N	7°52′14″ E	1	1
	Staldenried	46°13′49″ N	7°52′59″ E	3	43
	Täsch	46°04′01″ N	7°46′42″ E	1	26
	Törbel	46°14′16″ N	7°51′06″ E	6	96
	Visp	46°17′39″ N	7°52′56″ E	5	83
	Visperterminen	46°15′32″ N	7°54′09″ E	9	205
	Zeneggen	46°16′22″ N	7°51′58″ E	1	8
	Zermatt	46°01′11″ N	7°44′46″ E	4	40
Total				76 (27.44%)	1319 (32.06%)
Westlich Raron	Ausserberg	46°18′47″ N	7°50′57″ E	3	88
	Blatten(Lötschen)	46°25′13″ N	7°49′10″ E	1	34
	Bürchen	46°16′50″ N	7°48′56″ E	1	1
	Raron	46°18′41″ N	7°47′59″ E	2	22
	Steg	46°18′49″ N	7°44′56″ E	1	7
	Unterbäch	46°17′08″ N	7°47′50″ E	2	20
	Wiler (Lötschen)	46°24′15″ N	7°47′04″ E	3	87
Total				13 (4.69%)	259 (6.30%)
TOTAL	105			277 (100.00%)	4114 (100.00%)

^a^For each location, the following information is provided: name of the district, name of the location, latitude, longitude, number of flocks (n.flock), and the number of goat sera (n.sera) analyzed in the study

#### Absorption test and serum neutralization test

All ELISA-positive samples were tested with the absorption test (*n* = 175) and the serum neutralization test (*n* = 173; for two samples, one AT-positive and one AT-negative, there was not enough serum); results for individual goats are in Table S1 of Additional file 1. For the absorption test there were 50 positive, 6 equivocal and 117 negative serum samples. Thus, of the 175 serum samples, 28.6% (50/175) or 32.0% (56/175) tested positive on the AT depending on whether the 6 equivocal samples were treated as negative or positive. For the serum neutralization test, there were 70 positive and 103 negative serum samples. Thus, of the 173 serum samples, 40.4% (70/173) tested positive on the SNT. The 70 SNT-positive samples came from 26 different flocks of goats. To calculate the specificity and sensitivity of the absorption test, we used the serum neutralization test as the gold standard. When the 6 equivocal AT serum samples were classified as negative, the specificity of the absorption test was 100.0% and the sensitivity was 71.4%. When the 6 equivocal AT serum samples were classified as positive, the specificity was 98.1% and the sensitivity was 77.1% (Table 2). Of the 100 randomly selected goat sera, 97 were negative for both ELISA and SNT, one sample was negative for ELISA (just below the cut-off) but positive for SNT, and two samples were positive for both ELISA and SNT.

**Table 2:** Comparison between the serum neutralization test (SNT) and the absorption test (AT) for 173 goat serum samples that tested positive for TBEV-specific antibodies on a preliminary ELISA

	SNT positive	SNT negative	Total
AT positive	50	0	50
AT equivocal	4	2	6
AT negative	16	101	117
Total	70	103	173

The association of the TBEV-positive or TBEV-negative status was highly significant between the SNT and the AT (*p* < 0.001). The SNT is considered as the gold standard for deciding whether a goat was exposed to tick-borne encephalitis virus

#### Origin of goats

For 3653 animals belonging to 249 owners, the current locality of the owner and the locality from which the goats were purchased were known. For 2543 (69.6%) goats, the current locality and the locality of origin were the same and for the remaining 1110 goats (33.3%), these two localities were different. 3201 (87.6%) goats were born in the canton of Valais and the remaining 452 goats originated from 23 other Swiss cantons. Of the 452 goats born outside the canton of Valais, the majority (54.2%) came from the neighboring cantons of Bern, Fribourg and Vaud.

The geographical distribution map of TBEV-seropositive goats was created based on the results of serum neutralization test as this is the gold standard. Seropositive goats originating from other Swiss cantons were marked specifically, like the goat flock of Reckingen (Fig. 1, Table 3). Most of the seropositive goats were located in the known TBE risk area between the towns of Sierre and Visp. Other seropositive goats were found to the east of this area around the town of Brig and to the south of this area in the adjoining valley of St-Niklaus-Saas (Fig. 1). One flock of 26 goats, of which seven were positive for TBEV antibodies (according to the SNT), was discovered in the municipality of Finhaut, which is located 60 km west of the known TBEV-endemic area (Fig. 1).

**Table 3:** Seroprevalence of TBEV-specific IgG antibodies in seropositive goat flocks collected between October 2011 and March 2012 in the canton of Valais

District	Municipality of flock	Total # of sera	SNT-positive sera	SNT Sero-prev (%)	Canton of origin	Municipality of origin	TBEV-endemic area^a^
Brig	Glis	13	9	69.23	Valais	Glis	No
					Lucerne	Büron	Yes
	Mund	62	2	3.23	Bern	Zweisimmen	No
					Valais	Ausserberg	No
	Naters	30	6	20.00	Valais	Naters	No
					Valais	Glis	No
		25	1	4.00	Valais	Naters	No
	Ried-Brig	8	1	12.50	St-Gall	Salez	No
	Termen	6	1	16.67	Valais	Naters	No
Total		144	20	20.94			
Goms	Reckingen	99	5	5.05		Oberwil im Simmental	Yes
						Oensingen	Yes
						Bettlach	No
Total		99	5	5.05			
Hérens	Euseigne	51	1	1.96		Euseigne	No
Total		51	1	1.96			
Leuk	Ergisch	6	1	16.67		Ergisch	No
	Niedergampel	10	1	10.00		Niedergampel	No
	Susten	5	1	20.00		Niedergampel	No
	Susten	16	6	37.50		St-Niklaus	No
						Susten	No
						Turtmann	No
	Susten	12	10	83.33		Gonten	No
						Stalden	No
						Susten	No
	Turtmann	2	1	50.00		Susten	No
Total		51	20	36.25			
Monthey	Collombey	37	1	2.70		Bex	No
Total		37	1	2.70			
St-Maurice	Finhaut	26	7	26.92		Finhaut	No
Total		26	7	26.92			
Visp	Eisten	9	1	11.11		Eisten	No
	Saas-Grund	9	3	33.33		Blumenstein	Yes
						Saas-Grund	No
	St-Niklaus	3	1	33.33		St-Niklaus	No
		22	0	0.00			
		20	0	0.00			
	Staldenried	24	3	12.50		Stalden	No
	Visp	52	3	5.77		Glis	No
						Visp	No
	Visp	6	3	50.00		Unknown	
	Visperterminen	36	1	2.78		Visperterminen	No
		38	1	2.63		Visperterminen	No
Total		219	16	15.15			
TOTAL		627	70	11.16			

^a^Zones where TBE vaccination is recommended by the Federal Office of Public Health

For each seropositive goat flock, the following information is given: the district and the municipality where the flock is located, the total number of sera tested, the number of SNT-positive sera, the SNT seroprevalence (%), the canton and municipality from which the goats were originally obtained, and whether the site of origin was a TBEV-endemic area

#### Sex, age and breed of the goats

Individual information about the goats including origin, sex, age and breed were obtained for 1372 animals. Of these 1372 animals, 92.0% (1262/1372) were females and 8.0% (110/1372) were males. The age of the goats ranged from 0.5 to 11 years with a mean of 2.8 years (95% confidence limits (CL): 2.7–2.9 years). The most common goat breeds were col noir 63.1% (866/1372), chamoisée 21.5% (295/1373), Gessenay 5.4% (74/1372), and Grisonne 3.3% (45/1372). The other breeds (*n* = 92) included Boer, Verzasca, Toggenburg, Naine, Paon, Appenzell, Botée, and hybrids.

Of the 173 serum samples tested by SNT, the age of the goat was known for 115 individuals. The mean age of the seropositive goats (*n* = 52, mean = 3.4 years; 95% CL = 2.8–4.1 years) was almost twice as high as that of the seronegative goats (*n* = 63, mean = 1.6 years; 95% CL = 1.2–2.1 years) and this difference was statistically significant (χ^2^ = 29.85, df = 10, *p* = 0.0009). Of the 70 SNT-positive serum samples, the sex and breed of the goat were known for 52 individuals. Among the 52 SNT-positive goats, 51 were female and one was male. Of the 52 SNT-positive goats, 80.8% (42/52) were col noir, 9.6% (5/52) were Gessenay, 3.9% (2/52) were Appenzell, one was Chamoisée (1.9%) and 3.9% (2/52) were hybrids.

Sites of pasture were known for 24 flocks, the average distance between the pasture site and the owner’s place of residence was 3 km (95% CL = 1.9–4.1 km) and the mean number of pasture sites per owner was 2 (95% CL = 1.6–2.4 sites per owner).

As TBEV foci can be very small [9], the exact location of where goats were pastured was very important in cases where we sampled the local tick population for TBEV. For creating the maps, the nearby residences of the owners could be used because of the low average distance of 3 km.

### Ticks and TBEV

The three sites in the canton of Valais that had TBEV-seropositive flocks were located (1) near the town of Brig, (2) near the town of Gampel on the north side of the Rhone River, and (3) in an isolated area to the west of the municipality of Finhaut (Fig. 1). A total of 2045 *I. ricinus* ticks (adults and nymphs) were tested for TBEV using quantitative realtime RT-PCR [45]. TBEV-positive pools of ticks were detected at the site near Brig and the site near Gampel. With respect to the Gampel site, TBEV-positive pools of ticks were sampled from a pasture site that was located near Pletschen, which is 12 km from the town of Gampel and is located on the south side of the Rhone River. None of the tick pools from the two pasture sites near Finhaut tested positive for TBEV, despite a large sample size (1263 ticks).

## Discussion

The most important result was that we were able to detect two new TBEV foci out of three potential sites based on the initial detection of seropositive goats and the subsequent confirmation of TBEV-positive ticks [45, 46]. The identification of TBEV foci in *I. ricinus* tick populations is difficult for a number of reasons. The geographic distribution of TBEV foci is highly patchy, and the foci are often very small with an area of about 100 m^2^ [20, 21]. At such foci, the percentage of infected ticks is generally very low (<1%). Identification of new TBEV foci in the tick population therefore depends on strong a priori evidence that such a focus exists. Goats are interesting sentinel animals to use for sero-epidemiological surveys of TBEV. In Switzerland, goats are kept in the same two or three enclosed pasture sites year after year. If a TBEV focus is present, the goats will encounter infected ticks over the years and develop an antibody response. In summary, the fact that goats are kept in small, well-defined pasture sites in Switzerland makes them ideal sentinel hosts for screening for TBEV.

From an epidemiological point of view, the method used in the present study should be effective at identifying seropositive flocks without necessarily identifying every seropositive goat in that flock. In our study, we were confronted with some unexplained results. For example, one confirmed positive goat was detected in a flock of 51 goats in the village of Euseigne. Checking the origin of the seropositive goat confirmed that it was born and raised in Euseigne. This observation suggests that there may be a TBEV focus in the pasture sites of Euseigne even though this village is not located in the known endemic area of TBEV. An alternative explanation is that this serum sample produced a false positive on both the ELISA and the SNT, but such a result would be unlikely. As shown in a district in Thuringia, Germany, a single positive goat can provide evidence of a TBE risk area [38]. A sero-survey of the flock in Euseigne should be initiated in the future. Our study also showed the importance of knowing the origin of the goats. In a flock of goats in Reckingen, five sera tested positive, but all of these goats originated from the cantons of Bern and Solothurn, where TBEV is endemic. Thus, the conservative conclusion is that these goats were exposed to TBEV in these other cantons before being transported to the canton of Valais.

Statistical analyses are difficult due to the patchy pattern of TBEV foci and because in the present study, 50% of the goats originated from the known endemic area between Sierre and Visp. Data on sex and breed show that TBEV-positive goats are typical of the ‘average’ goat in the canton of Valais. Most of the goats in the canton of Valais are female (92.0% = 1262/1372) and most belong to the col noir breed (63.1% = 866/1372). Similarly, most of the TBEV-positive goats in the present study were female (51/52) and belonged to the col noir breed (80.8% = 42/52). This breed of goats is specific to the German-speaking region, which includes the known TBEV foci, and we experienced some difficulties in obtaining complete data from some of these goat flocks. It is unlikely that a TBEV infection is associated with a specific breed or sex. In contrast, breed is important in dogs where individuals with long and pale hair were more often infected by TBEV [48]. Seroprevalence increased with age as for many other infectious diseases. The mean age of the 52 positive goats was 3.4 years compared to the mean age of 2.8 years for the entire sample of goats. These findings were in agreement with other studies on sheep [47], where older animals with more than one season on the pasture were more likely to have TBEV antibodies than younger animals. The obvious explanation is that older animals are more likely to have been exposed to TBEV-infected ticks than younger animals.

The design of our ELISA allowed for quick screening of >4000 individual goat serum samples. A subsample of 175 of the most reactive goat serum samples was studied further using the absorption test (AT) and the serum neutralization test (SNT). Of the subsample of goat serum samples that were highly reactive on the ELISA, 26.9–33.1% tested positive on the AT (depending on how the equivocal samples were treated) and 40.4% tested positive on the SNT. Comparison between the AT and the SNT revealed that the AT was highly specific (98.1–100.0%), but with moderate sensitivity (71.4–77.1%; Table 2). The testing of 100 randomly selected serum samples by SNT demonstrated that the results of our ELISA were good. ELISA is the best and easiest assay to screen a large number of sera. Positive sera need to be checked using the gold standard SNT to confirm TBEV-seropositive status. A possible explanation for serum samples that test positive on the ELISA but negative on the SNT is infection with other flaviviruses that cross-react with the antigens of the ELISA assay. Possible flaviviruses that could result in cross-reactivity include the louping ill virus and the “ruminant” TBEV-like viruses that have been identified recently [49, 50].

The tick-borne encephalitis virus is classified into three subtypes that are found in different geographical areas: European (TBEV-Eu), Siberian (TBEV-Sib), and Far Eastern (TBEV-FEa) [11, 51, 52]. The European subtype is the least virulent subtype and is responsible for all human cases of TBE in Western Europe. In Switzerland, TBEV isolates circulating in ticks collected at 39 foci were closely related and all of them belonged to the European subtype [53]. In our previous study, we had shown that all tick-derived TBEV isolates from the canton of Valais belonged to the European subtype [45]. In vivo and in vitro studies of Swiss TBEV isolates suggest a high number of avirulent isolates, which is in agreement with a high proportion of subclinical or mild TBE infections in the Swiss public [53].

## Conclusions

Our present study confirmed the utility of goats as sentinel animals in sero-epidemiological surveys of TBEV. Sero-surveys help to identify candidate sites where ticks should be collected to detect new TBEV foci. ELISA is an effective and simple tool to screen large numbers of sentinel hosts for antibodies against TBEV. Confirmation of ELISA-positive results is necessary and the SNT is the recommended gold standard. Knowledge of the origin of seropositive goats is essential to further define the potential presence of a new TBEV focus. Our sero-survey of goats in the canton of Valais revealed several hot spots that should be investigated in the future.

## Additional file

Additional file 1: Table S1. Summary of results obtained from ELISA, absorption test and serum neutralization test for 173 goat sera. This table contains all the results from the ELISA test, the absorption test and the serum neutralization test of the goat sera included in the manuscript. (DOCX 28 kb)

## Abbreviations

AT: absorption test
BHK: baby hamster kidney
BSA: bovine serum albumin
CAE: caprine arthritis encephalitis
ELISA: enzyme - linked immunosorbent assay
Er: extinction rate
FOPH: Swiss Federal Office for Public Health (english name for OFSP)
I: *Ixodes*
ICHV: Institut Central des Hôpitaux de Valais
Ig: immunoglobulin
ND: neutralizing dose
OD: optical density
OFSP: Office fédéral de la santé publique (french name for FOPH)
RT-PCR: reverse transcriptase polymerase chain reaction
SFGR: Swiss Federation of Goat Rearing
SNT: serum neutralization test
TBE: tick-borne encephalitis
TBEV: tick-borne encephalitis virus
TBS: Tris-buffered saline solution
TCID: tissue culture infectious dose
