# Supplementary material for: Goats as sentinel hosts for the detection of tick-borne encephalitis risk areas in the Canton of Valais, Switzerland
Source: BMC Vet Res. 2017 Jul 11;13:217. doi: 10.1186/s12917-017-1136-y (PMC5504567; doi:10.1186/s12917-017-1136-y)
Supplement: Additional file 1: Table S1. — Summary of results obtained from ELISA, absorption test and serum neutralization test for 173 goat sera. This table contains all the results from the ELISA test, the absorption test and the serum neutralization test of the goat sera included in the manuscript. (DOCX 28 kb) [file 12917_2017_1136_MOESM1_ESM.docx]

Additional file 1: Table S1: Summary of results obtained from Elisa, absorption test and SNT for 173 goat sera

|  |  |  |  | ELISA 2 | SNT | | ABSORPTION TEST | | | |
| --- | --- | --- | --- | --- | --- | --- | --- | --- | --- | --- |
|  | identification number of the goat |  |  | DO | value | rating | DO normal | DO absorbed | extinction % | rating |
| 1 | 2120131 | -020 | XC01 | 1.501 | 5 | Neg | 0.27 | 0.27 | -1.12 | Neg |
| 2 | 2120203 | -019 | XC03 | 1.817 | >960 | Pos | 0.73 | 0.22 | 69.44 | Pos |
| 3 | 2120203 | -019 | XC05 | 1.649 | 480 | Pos | 0.33 | 0.06 | 81.74 | Pos |
| 4 | 2120203 | -019 | XC06 | 1.482 | 120 | Pos | 0.24 | 0.06 | 75.31 | Pos |
| 5 | 2120203 | -019 | XC07 | 1.167 | 15 | Pos | 0.20 | 0.15 | 25.76 | Neg |
| 6 | 2120203 | -020 | XC01 | 1.462 | 60 | Pos | 0.25 | 0.08 | 69.84 | Pos |
| 7 | 2120203 | -020 | XC05 | 1.73 | 45 | Pos | 0.67 | 0.25 | 63.38 | Pos |
| 8 | 2120203 | -020 | XC07 | 1.46 | 240 | Pos | 0.29 | 0.09 | 68.64 | Pos |
| 9 | 2120107 | -038 | XC02 | 0.733 | <5 | Neg | 0.23 | 0.26 | -14.41 | Neg |
| 10 | 2120107 | -038 | XC05 | 0.981 | 22.5 | Pos | 0.09 | 0.03 | 71.11 | Pos |
| 11 |  |  | CL1 | 1.455 | 90 | Pos | 0.25 | 0.07 | 73.41 | Pos |
| 12 |  |  | CL5 | 1.821 | >960 | Pos | 0.66 | 0.08 | 88.69 | Pos |
| 13 |  |  | CL6 | 1.231 | 10 | Pos | 0.28 | 0.26 | 7.61 | Neg |
| 14 | 2120214 | -025 | XC07 | 1.585 | 5 | Neg | 0.57 | 0.43 | 24.42 | Neg |
| 15 | 2111213 | -037 | XC10 | 1.297 | <5 | Neg | 0.50 | 0.52 | -4.41 | Neg |
| 16 | 2111214 | -022 | XC02 | 1.621 | 180 | Pos | 0.30 | 0.09 | 71.43 | Pos |
| 17 | 2111214 | -022 | XC10 | 1.441 | <5 | Neg | 0.40 | 0.31 | 22.28 | Neg |
| 18 | 2111217 | -016 | XC02 | 1.558 | 15 | Pos | 0.33 | 0.18 | 44.85 | Neg |
| 19 | 2111217 | -016 | XC03 | 1.615 | 360 | Pos | 0.34 | 0.06 | 82.22 | Pos |
| 20 | 2111217 | -016 | XC07 | 1.781 | >960 | Pos | 0.61 | 0.14 | 77.30 | Pos |
| 21 | 2111217 | -016 | XC08 | 1.548 | 480 | Pos | 0.23 | 0.05 | 80.60 | Pos |
| 22 | 2111217 | -016 | XC10 | 1.466 | 30 | Pos | 0.25 | 0.15 | 39.53 | Neg |
| 23 | 2111217 | -016 | XC12 | 1.372 | 180 | Pos | 0.21 | 0.05 | 77.73 | Pos |
| 24 | 2111220 | -006 | XC06 | 1.259 | <5 | Neg | 0.57 | 0.59 | -4.21 | Neg |
| 25 | 2111220 | -008 | XC05 | 1.453 | <5 | Neg | 0.64 | 0.58 | 8.79 | Neg |
| 26 | 2111220 | -012 | XC02 | 1.474 | 180 | Pos | 0.34 | 0.05 | 86.39 | Pos |
| 27 | 2111220 | -013 | XC02 | 1.483 | 60 | Pos | 0.29 | 0.14 | 51.58 | Equivocal |
| 28 | 2111221 | -013 | XC05 | 1.42 | <7.5 | Neg | 0.63 | 0.64 | -2.23 | Neg |
| 29 | 2111221 | -014 | XC10 | 1.395 | <7.5 | Neg | 0.33 | 0.29 | 12.91 | Neg |
| 30 | 2111228 | -035 | XC10 | 1.437 | 90 | Pos | 0.19 | 0.05 | 72.16 | Pos |
| 31 | 2111228 | -036 | XC02 | 1.254 | <5 | Neg | 0.43 | 0.44 | -3.04 | Neg |
| 32 | 2111228 | -036 | XC04 | 1.18 | <5 | Neg | 0.28 | 0.31 | -7.39 | Neg |
| 33 | 2111228 | -036 | XC11 | 1.406 | <7.5 | Neg | 0.82 | 0.82 | 0.00 | Neg |
| 34 | 2111230 | -005 | XC02 | 1.47 | <5 | Neg | 0.67 | 0.76 | -12.80 | Neg |
| 35 | 2111230 | -005 | XC09 | 1.251 | 120 | Pos | 0.19 | 0.05 | 76.68 | Pos |
| 36 | 2120104 | -016 | XC01 | 1.233 | <5 | Neg | 0.23 | 0.26 | -10.39 | Neg |
| 37 | 2120106 | -008 | XC03 | 1.377 | 45 | Pos | 0.24 | 0.06 | 76.45 | Pos |
| 38 | 2120106 | -009 | XC01 | 1.261 | 60 | Pos | 0.23 | 0.06 | 73.33 | Pos |
| 39 | 2120106 | -012 | XC05 | 1.436 | 60 | Pos | 0.28 | 0.10 | 62.45 | Pos |
| 40 | 2120112 | -029 | XC09 | 1.573 | 90 | Pos | 0.32 | 0.08 | 76.25 | Pos |
| 41 | 2120112 | -030 | XC04 | 1.14 | 720 | Pos | 0.14 | 0.02 | 83.33 | Pos |
| 42 | 2120112 | -030 | XC06 | 1.309 | 720 | Pos | 0.19 | 0.03 | 84.54 | Pos |
| 43 | 2120112 | -033 | XC06 | 1.476 | <7.5 | Neg | 0.29 | 0.37 | -29.12 | Neg |
| 44 | 2120113 | -023 | XC09 | 1.226 | <5 | Neg | 0.30 | 0.32 | -8.47 | Neg |
| 45 | 2120113 | -025 | XC02 | 1.279 | 480 | Pos | 0.25 | 0.08 | 69.72 | Pos |
| 46 | 2120113 | -028 | XC11 | 1.175 | <5 | Neg | 0.23 | 0.26 | -12.23 | Neg |
| 47 | 2120113 | -035 | XC05 | 1.609 | <7.5 | Neg | 0.50 | 0.38 | 23.69 | Neg |
| 48 | 2120113 | -038 | XC01 | 1.392 | 30 | Pos | 0.22 | 0.06 | 71.75 | Pos |
| 49 | 2120119 | -021 | XC01 | 1.518 | <7.5 | Neg | 0.48 | 0.41 | 15.29 | Neg |
| 50 | 2120120 | -014 | XC04 | 1.571 | 22.5 | Pos | 0.27 | 0.13 | 53.65 | Equivocal |
| 51 | 2120120 | -014 | XC07 | 1.371 | 480 | Pos | 0.19 | 0.05 | 73.80 | Pos |
| 52 | 2120120 | -014 | XC09 | 1.038 | 30 | Pos | 0.11 | 0.05 | 57.80 | Pos |
| 53 | 2120120 | -014 | XC10 | 1.452 | 60 | Pos | 0.24 | 0.06 | 76.79 | Pos |
| 54 | 2120120 | -015 | XC05 | 0.933 | 22.5 | Pos | 0.11 | 0.05 | 55.45 | Pos |
| 55 | 2120120 | -015 | XC06 | 0.927 | 30 | Pos | 0.12 | 0.06 | 52.17 | Equivocal |
| 56 | 2120210 | -014 | XC03 | 1.474 | <7.5 | Neg | 0.46 | 0.52 | -13.63 | Neg |
| 57 | 2120210 | -014 | XC07 | 1.351 | <5 | Neg | 0.32 | 0.31 | 3.73 | Neg |
| 58 | 211118 | -006 | XC10 | 0.754 | <5 | Neg | 0.18 | 0.20 | -8.74 | Neg |
| 59 | 2111124 | -010 | XC02 | 1.746 | <7.5 | Neg | 0.63 | 0.63 | 0.32 | Neg |
| 60 | 2111124 | -012 | XC10 | 0.993 | 5 | Neg | 0.18 | 0.18 | -1.67 | Neg |
| 61 | 2111121 | -003 | XC02 | 1.124 | <5 | Neg | 0.16 | 0.08 | 53.09 | Equivocal |
| 62 | 2111125 | -003 | XC10 | 1.181 | <5 | Neg | 0.22 | 0.23 | -2.69 | Neg |
| 63 | 2111125 | -004 | XC03 | 1.057 | <5 | Neg | 0.20 | 0.20 | 1.50 | Neg |
| 64 | 2111125 | -004 | XC07 | 1.612 | <5 | Neg | 0.45 | 0.47 | -3.10 | Neg |
| 65 | 2111125 | -005 | XC08 | 1.101 | <5 | Neg | 0.20 | 0.21 | -4.57 | Neg |
| 66 | 2111125 | -006 | XC02 | 1.156 | <5 | Neg | 0.57 | 0.61 | -7.01 | Neg |
| 67 | 2111129 | -038 | XC07 | 1.287 | <5 | Neg | 0.32 | 0.31 | 5.57 | Neg |
| 68 | 2111129 | -040 | XC10 | 0.976 | <5 | Neg | 0.24 | 0.27 | -14.41 | Neg |
| 69 | 2111129 | -041 | XC07 | 1.223 | <5 | Neg | 0.35 | 0.29 | 17.56 | Neg |
| 70 | 2111130 | -012 | XC09 | 1.543 | <5 | Neg | 0.42 | 0.51 | -19.95 | Neg |
| 71 | 2111130 | -013 | XC05 | 1.019 | <5 | Neg | 0.17 | 0.16 | 4.82 | Neg |
| 72 | 2111130 | -014 | XC05 | 1.379 | <5 | Neg | 0.41 | 0.37 | 9.66 | Neg |
| 73 | 2111130 | -016 | XC08 | 1.305 | <5 | Neg | 0.29 | 0.32 | -10.27 | Neg |
| 74 | 2111130 | -020 | XC07 | 1.131 | 5 | Neg | 0.34 | 0.23 | 33.23 | Neg |
| 75 | 2111202 | -008 | XC03 | 0.903 | <5 | Neg | 0.29 | 0.33 | -13.84 | Neg |
| 76 | 2111202 | -009 | XC04 | 1.474 | <7.5 | Neg | 0.26 | 0.29 | -14.45 | Neg |
| 77 | 2111202 | -009 | XC06 | 0.966 | <5 | Neg | 0.20 | 0.23 | -18.46 | Neg |
| 78 | 2111202 | -022 | XC01 | 1.022 | 120 | Pos | 0.11 | 0.03 | 71.93 | Pos |
| 79 | 2111202 | -022 | XC02 | 1.088 | 7.5 | Pos | 0.14 | 0.09 | 32.86 | Neg |
| 80 | 2111202 | -022 | XC03 | 1.176 | 22.5 | Pos | 0.15 | 0.06 | 59.60 | Pos |
| 81 | 2111202 | -024 | XC03 | 1.101 | <5 | Neg | 0.17 | 0.09 | 45.61 | Equivocal |
| 82 | 2111202 | -029 | XC09 | 1.147 | <5 | Neg | 0.30 | 0.33 | -11.71 | Neg |
| 83 | 2111203 | -004 | XC06 | 1.704 | <7.5 | Neg | 0.65 | 0.70 | -8.36 | Neg |
| 84 | 2111206 | -011 | XC03 | 1.468 | >960 | Pos | 0.27 | 0.05 | 81.95 | Pos |
| 85 | 2111206 | -011 | XC10 | 1.382 | 120 | Pos | 0.23 | 0.05 | 77.29 | Pos |
| 86 | 2111206 | -012 | XC10 | 1.195 | 480 | Pos | 0.16 | 0.03 | 78.98 | Pos |
| 87 | 2111206 | -013 | XC05 | 1.447 | 180 | Pos | 0.28 | 0.10 | 65.84 | Pos |
| 88 | 2111206 | -014 | XC01 | 1.454 | 240 | Pos | 0.28 | 0.12 | 55.87 | Pos |
| 89 | 2111206 | -032 | XC05 | 0.949 | <5 | Neg | 0.18 | 0.18 | -0.57 | Neg |
| 90 | 2111207 | -022 | XC08 | 0.975 | <5 | Neg | 0.21 | 0.21 | -3.88 | Neg |
| 91 | 2111207 | -023 | XC04 | 1.127 | <5 | Neg | 0.22 | 0.24 | -12.50 | Neg |
| 92 | 2111208 | -028 | XC02 | 1.524 | <5 | Neg | 0.86 | 0.79 | 8.92 | Neg |
| 93 | 2111208 | -029 | XC03 | 1.72 | <7.5 | Neg | 0.61 | 0.50 | 18.60 | Neg |
| 94 | 2111208 | -030 | XC02 | 1.249 | <5 | Neg | 0.22 | 0.24 | -9.26 | Neg |
| 95 | 2111208 | -031 | XC02 | 1.269 | <5 | Neg | 0.30 | 0.28 | 6.44 | Neg |
| 96 | 2111209 | -007 | XC02 | 1.735 | <7.5 | Neg | 0.89 | 0.96 | -6.94 | Neg |
| 97 | 2111210 | -013 | XC01 | 0.989 | <5 | Neg | 0.12 | 0.13 | -11.97 | Neg |
| 98 | 2111213 | -032 | XC08 | 0.971 | <5 | Neg | 0.15 | 0.15 | 1.36 | Neg |
| 99 | 2111213 | -033 | XC07 | 1.368 | 7.5 | Pos | 0.31 | 0.38 | -20.90 | Neg |
| 100 | 2111213 | -033 | XC09 | 0.837 | <5 | Neg | 0.28 | 0.32 | -13.52 | Neg |
| 101 | 2111213 | -033 | XC10 | 0.913 | <5 | Neg | 0.10 | 0.11 | -13.00 | Neg |
| 102 | 2111213 | -034 | XC10 | 1.543 | 10 | Pos | 0.66 | 0.59 | 11.21 | Neg |
| 103 | 2111213 | -035 | XC04 | 0.902 | <5 | Neg | 0.11 | 0.09 | 16.07 | Neg |
| 104 | 2111213 | -035 | XC09 | 0.869 | <5 | Neg | 0.30 | 0.32 | -6.02 | Neg |
| 105 | 2111213 | -040 | XC04 | 1.413 | 180 | Pos | 0.21 | 0.05 | 77.67 | Pos |
| 106 | 2111101 | -018 | XC01 | 1.302 | <5 | Neg | 0.38 | 0.47 | -24.27 | Neg |
| 107 | 2111101 | -018 | XC05 | 0.945 | <5 | Neg | 0.26 | 0.33 | -24.90 | Neg |
| 108 | 2111101 | -021 | XC03 | 0.814 | <5 | Neg | 0.24 | 0.26 | -6.97 | Neg |
| 109 | 2111026 | -017 | XC11 | 1.668 | <7.5 | Neg | 0.66 | 0.54 | 18.29 | Neg |
| 110 | 2111026 | -018 | XC03 | 1.041 | <5 | Neg | 0.15 | 0.18 | -26.90 | Neg |
| 111 | 2111026 | -021 | XC07 | 1.163 | <5 | Neg | 0.21 | 0.18 | 15.57 | Neg |
| 112 | 2111107 | -004 | XC01 | 0.791 | <5 | Neg | 0.18 | 0.13 | 26.52 | Neg |
| 113 | 2111109 | -039 | XC05 | 1.351 | <7.5 | Neg | 0.26 | 0.25 | 6.82 | Neg |
| 114 | 2111111 | -003 | XC01 | 1.346 | <7.5 | Neg | 0.44 | 0.52 | -19.50 | Neg |
| 115 | 2111111 | -006 | XC02 | 1.57 | <5 | Neg | 0.56 | 0.46 | 18.00 | Neg |
| 116 | 2111114 | -002 | XC05 | 0.806 | 60 | Pos | 0.19 | 0.21 | -13.98 | Neg |
| 117 | 2111114 | -002 | XC07 | 1.016 | <5 | Neg | 0.27 | 0.39 | -46.07 | Neg |
| 118 | 2111115 | -014 | XC02 | 1.003 | <5 | Neg | 0.26 | 0.30 | -16.60 | Neg |
| 119 | 2111115 | -014 | XC03 | 1.28 | <5 | Neg | 0.62 | 0.69 | -11.20 | Neg |
| 120 | 2111115 | -014 | XC04 | 0.851 | 5 | Neg | 0.36 | 0.43 | -20.79 | Neg |
| 121 | 2111117 | -017 | XC10 | 1.069 | <5 | Neg | 0.23 | 0.28 | -18.88 | Neg |
| 122 | 2111117 | -019 | XC08 | 1.544 | <7.5 | Neg | 0.38 | 0.41 | -7.57 | Neg |
| 123 | 2111117 | -024 | XC03 | 1.528 | <7.5 | Neg | 0.57 | 0.56 | 1.05 | Neg |
| 124 | 2111118 | -013 | XC01 | 1.565 | >480 | Pos | 0.37 | 0.10 | 72.60 | Pos |
| 125 | 2111118 | -013 | XC02 | 1.275 | 60 | Pos | 0.20 | 0.05 | 76.50 | Pos |
| 126 | 2111118 | -013 | XC03 | 1.002 | 22.5 | Pos | 0.11 | 0.03 | 76.11 | Pos |
| 127 | 2111118 | -013 | XC04 | 1.324 | 90 | Pos | 0.22 | 0.04 | 83.04 | Pos |
| 128 | 2111118 | -013 | XC06 | 1.537 | 180 | Pos | 0.36 | 0.09 | 75.35 | Pos |
| 129 | 2111118 | -013 | XC08 | 1.543 | 22.5 | Pos | 0.29 | 0.21 | 25.26 | Neg |
| 130 | 2111118 | -013 | XC09 | 0.817 | 90 | Pos | 0.13 | 0.06 | 52.76 | Equivocal |
| 131 | 2111118 | -013 | XC10 | 1.457 | 30 | Pos | 0.28 | 0.22 | 21.83 | Neg |
| 132 | 2111118 | -013 | XC11 | 1.518 | 120 | Pos | 0.37 | 0.10 | 73.15 | Pos |
| 133 | 2111118 | -013 | XC12 | 0.953 | 30 | Pos | 0.10 | 0.04 | 58.16 | Pos |
| 134 | 2111118 | -015 | XC07 | 1.048 | <5 | Neg | 0.18 | 0.20 | -10.06 | Neg |
| 135 | 2111119 | -011 | XC06 | 1.709 | <7.5 | Neg | 0.88 | 0.88 | -0.11 | Neg |
| 136 | 2111119 | -012 | XC06 | 1.35 | <5 | Neg | 0.28 | 0.31 | -8.45 | Neg |
| 137 | 2111122 | -015 | XC06 | 1.259 | <5 | Neg | 0.19 | 0.22 | -13.16 | Neg |
| 138 | 2111122 | -019 | XC01 | 1.272 | <5 | Neg | 0.42 | 0.43 | -3.36 | Neg |
| 139 | 2111123 | -010 | XC02 | 0.96 | <5 | Neg | 0.22 | 0.25 | -13.76 | Neg |
| 140 | 2111123 | -010 | XC06 | 1.045 | <5 | Neg | 0.32 | 0.38 | -21.27 | Neg |
| 141 | 211118 | -006 | XC02 | 1.6 | <5 | Neg | 0.89 | 0.84 | 5.64 | Neg |
| 142 | 2111020 | -022 | XC07 | 1.445 | <5 | Neg | 0.55 | 0.55 | 0.00 | Neg |
| 143 | 2111020 | -031 | XC04 | 0.935 | <5 | Neg | 0.39 | 0.44 | -12.89 | Neg |
| 144 | 2111020 | -039 | XC06 | 1.165 | 22.5 | Pos | 0.36 | 0.36 | -1.97 | Neg |
| 145 | 2111020 | -041 | XC08 | 1.254 | <5 | Neg | 0.46 | 0.43 | 6.80 | Neg |
| 146 | 2111020 | -044 | XC06 | 1.208 | 7.5 | Pos | 0.55 | 0.60 | -8.30 | Neg |
| 147 | 2111020 | -044 | XC10 | 1.076 | 7.5 | Pos | 0.51 | 0.50 | 0.20 | Neg |
| 148 | 2111020 | -044 | XC12 | 1.202 | 7.5 | Pos | 0.19 | 0.21 | -9.52 | Neg |
| 149 | 2111021 | -007 | XC03 | 1.451 | <7.5 | Neg | 0.57 | 0.58 | -0.35 | Neg |
| 150 | 2111024 | -002 | XC04 | 1.236 | 5 | Neg | 0.35 | 0.30 | 15.06 | Neg |
| 151 | 2111024 | -004 | XC14 | 1.032 | <5 | Neg | 0.24 | 0.25 | -6.33 | Neg |
| 152 | 2111025 | -018 | XC01 | 1.591 | <7.5 | Neg | 0.55 | 0.57 | -4.94 | Neg |
| 153 | 2111025 | -018 | XC14 | 1.27 | 5 | Neg | 0.33 | 0.28 | 13.80 | Neg |
| 154 | 2120221 | -015 | XC04 | 0.834 | <5 | Neg | 0.15 | 0.16 | -8.67 | Neg |
| 155 | 2120221 | -016 | XC12 | 1.467 | 5 | Neg | 0.26 | 0.19 | 26.82 | Neg |
| 156 | 2120225 | -018 | XC05 | 0.919 | <5 | Neg | 0.34 | 0.32 | 7.62 | Neg |
| 157 | 2120225 | -019 | XC03 | 1.662 | <7.5 | Neg | 0.78 | 0.79 | -1.67 | Neg |
| 158 | 2120228 | -020 | XC02 | 1.745 | <5 | Neg | 0.49 | 0.35 | 27.90 | Neg |
| 159 | 2120210 | -016 | XC05 | 1.861 | <7.5 | Neg | 0.42 | 0.32 | 22.30 | Neg |
| 160 | 2111214 | -024 | XC06 | 1.156 | <5 | Neg | 0.18 | 0.17 | 1.14 | Neg |
| 161 | 2111214 | -025 | XC01 | 1.189 | 5 | Neg | 0.42 | 0.45 | -5.67 | Neg |
| 162 | 2111214 | -025 | XC03 | 1.065 | <5 | Neg | 0.76 | 0.81 | -6.58 | Neg |
| 163 | 2111216 | -013 | XC02 | 1.252 | 120 | Pos | 0.13 | 0.03 | 76.15 | Pos |
| 164 | 2111217 | -016 | XC04 | 1.224 | 120 | Pos | 0.10 | 0.03 | 72.12 | Pos |
| 165 | 2111217 | -016 | XC05 | 1.362 | 15 | Pos | 0.25 | 0.24 | 2.01 | Neg |
| 166 | 2111217 | -016 | XC06 | 1.334 | 180 | Pos | 0.16 | 0.09 | 44.30 | Neg |
| 167 | 2111220 | -007 | XC07 | 1.118 | <5 | Neg | 0.70 | 0.75 | -7.41 | Neg |
| 168 | 2111220 | -011 | XC04 | 1.110 | 45 | Pos | 0.13 | 0.04 | 70.45 | Pos |
| 169 | 2111220 | -012 | XC03 | 1.214 | 120 | Pos | 0.15 | 0.06 | 61.22 | Pos |
| 170 | 2111220 | -012 | XC06 | 1.103 | 720 | Pos | 0.15 | 0.06 | 57.62 | Pos |
| 171 | 2111220 | -012 | XC10 | 1.239 | 60 | Pos | 0.14 | 0.06 | 57.55 | Pos |
| 172 | 2111221 | -017 | XC02 | 1.180 | <5 | Neg | 0.44 | 0.49 | -11.44 | Neg |
| 173 | 2120113 | -038 | XC07 | 1.113 | <5 | Neg | 0.15 | 0.10 | 33.33 | Neg |
